# Impact on arsenic exposure of a growing proportion of untested wells in Bangladesh

**DOI:** 10.1186/1476-069X-11-7

**Published:** 2012-02-21

**Authors:** Christine Marie George, Joseph H Graziano, Jacob L Mey, Alexander van Geen

**Affiliations:** 1 Department of Environmental Health Sciences, Mailman School of Public Health, Columbia University, New York, NY, USA; 2 Lamont-Doherty Earth Observatory of Columbia University, Palisades, New York, USA

**Keywords:** Arsenic, Field kit, Well screening, Bangladesh

## Abstract

**Background:**

In many areas of Bangladesh, it has been more than six years since a national campaign to test tubewells for arsenic (As) was conducted. Many households therefore draw their water for drinking and cooking from untested wells.

**Methods:**

A household drinking water survey of 6646 households was conducted in Singair upazilla of Bangladesh. A subset of 795 untested wells used by 1000 randomly selected households was tested in the field by trained village workers with the Hach EZ kit, using an extended reaction time of 40 min, and in the laboratory by high-resolution inductively-coupled plasma-mass spectrometry (HR ICP-MS).

**Results:**

The household survey shows that more than 80% of the wells installed since the national testing campaign in this area were untested. Less than 13% of the households with untested wells knew where a low-As well was located near their home. Village workers using the Hach EZ kit underestimated the As content of only 4 out of 795 wells relative to the Bangladesh standard. However, the As content of 168 wells was overestimated relative to the same threshold.

**Conclusion:**

There is a growing need for testing tubewells in areas of Bangladesh where As concentrations in groundwater are elevated. This could be achieved by village workers trained to use a reliable field kit. Such an effort would result in a considerable drop in As exposure as it increases the opportunities for well switching by households.

## Background

Elevated exposure to inorganic arsenic (As) is associated with cancers of the skin, bladder, and lung [1-3], reproductive and developmental effects [4,5], cardiovascular disease [6,7], and skin lesions [8,9]. In Bangladesh, millions of people are exposed to naturally occurring As concentrations that exceed the World Health Organization (WHO) guideline of 10 μg/L [10]. During the 1970s, the United Nations Children's Fund, through the government of Bangladesh, promoted the installation of tubewells to reduce risks from drinking microbial contaminated surface water [11]. In the early 1990s, evidence began to emerge that Bangladeshi villagers were presenting signs of arsenicosis due to the consumption of well water with elevated levels of As [12]. An As testing campaign relying on field kits and targeting 5 million wells in regions identified to be at risk for As contamination was initiated in 2001 and completed in 2004. By 2005, 1.4 million tubewells were found to have levels of As above the Bangladesh standard of 50 μg/L and were painted red, while another 3.5 million wells were found to be below the standard and were painted green [10]. It is estimated that approximately 12% of households presently drink water in Bangladesh that does not meet the Bangladesh standard for As [13].

The impact of As mitigation in Bangladesh, though significant, has been limited to a variety of approaches that currently serve roughly half of the affected population. The most common As mitigation option followed in rural areas has been well switching [10]. This involves switching from an As contaminated well to a nearby well that is safe relative to the Bangladesh standard for As in drinking water. Because of the spatial heterogeneity of As in groundwater well switching has been estimated to be a viable option for reducing exposure for all but 13% percent of the population that lives in areas with greater than 80% arsenic contamination [10,14]. Testing well water for As has been shown to reduce As exposure in villages of Bangladesh due to well switching on the basis of household surveys as well as urinary As measurements [15-17].

In this contribution, we report the results of two phases of a study conducted in Singair upazilla (subdistrict) of Bangladesh: 1) a sizeable household drinking water survey paired with the collection of geographic data; and 2) testing of a subset of wells of unknown status with a field kit by trained village workers as well as laboratory measurements. The household drinking water survey was conducted to determine the status of wells used six years after a blanket testing campaign for As swept through the area.

## Methods

### Sampling design

The study was conducted in rural Singair upazilla, located in Manikganj district of Bangladesh. This study area was selected on the basis of an expected wide range of As concentrations and the presence of the Christian Commission for Development Bangladesh (CCDB), a non-governmental organization that assisted with the implementation of this intervention. The first phase of the study was a household drinking water survey conducted in 26 villages; this survey did not involve well testing. The second phase of the study was an As testing intervention in which village workers conducted field As measurements for 1000 randomly selected study households using a well of unknown status.

**Figure 1:**
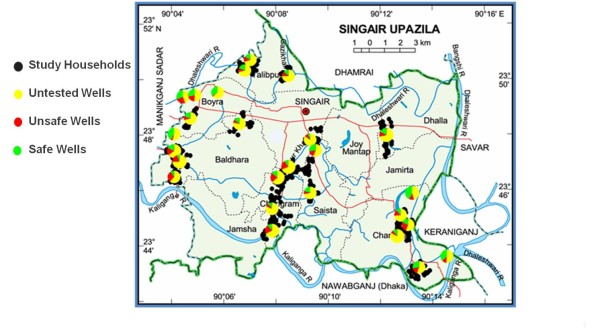
**Map of study area. Pie charts represent the proportion of untested, safe, and unsafe wells based on household recollection in 26 villages**. Black dots indicate the location of 1000 study households with an untested well randomly selected from a subset of 20 out of 26 villages where the household drinking water survey was conducted

The household drinking water survey was administered to all 6646 households in the 26 villages that could be contacted from November 2009 to January 2010 (Figure 1). Villages with at least 40% of wells exceeding the Bangladesh As standard (50 μg/L) were selected using data from the Bangladesh Arsenic Mitigation Water Supply Project (BAMWSP). Interviewers were sent to every household present in each of the villages to administer the survey questionnaire to the person in the household responsible for primary drinking water collection. For each household, the survey obtained information on the As status of the household's primary drinking water source, the well depth, well installation date, and if the well was painted based on the As concentration. For a subset of 10 villages, the position of each well was determined with hand-held global positioning system (GPS) receivers within an estimated accuracy of ~10 m. Because a typical private well is shared by several households, two on average, information was recorded repeatedly for a significant number of wells.

**Figure 2:**
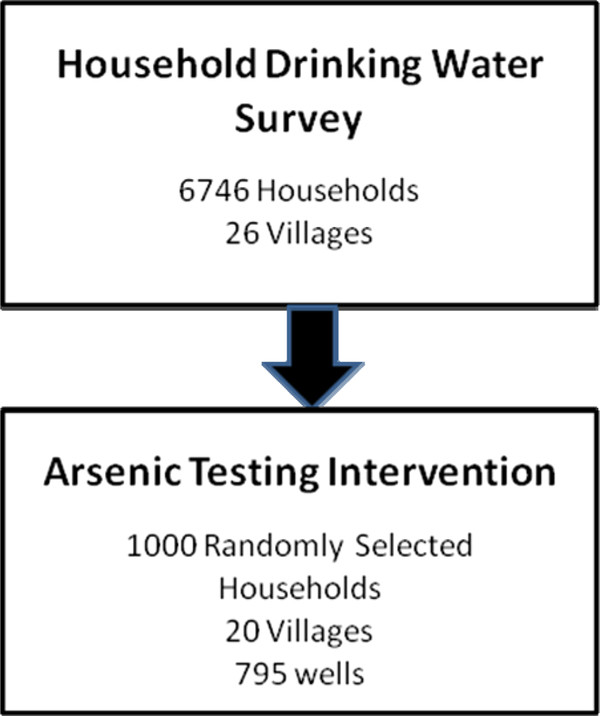
**Study design of household drinking water survey and arsenic testing intervention in Singair, Bangladesh**.

In the second phase of our study, a subset of 20 villages meeting our study eligibility criteria of having at least 40% of wells exceeding the Bangladesh As standard (50 μg/L), and at least 50 individuals who met the study eligibility criteria using the results of our household drinking water survey were selected to be part of an As testing intervention that was conducted from March to June 2010 (Figure 2). Based on a village census created from the household drinking water survey, 50 households with untested wells were selected at random from each of the 20 villages. Thus, the total study population was 1000 households. The primary drinking water source for each selected household was tested for As by village workers trained as part of the project. Because some of the 1000 study households shared the same tubewell, this survey covers only 795 previously untested tubewells distributed across the 20 study villages.

Twenty village workers were selected to conduct the As testing intervention by CCDB. They were a convenience sample, selected based on their ability to complete a reading and writing test. Their educational level ranged from completion of secondary school certificate to higher secondary school certificate (Grades 8-13). None had previous laboratory experience or prior experience using this field testing kit. Each of these village workers were trained to use the Hach EZ kit (Part No. 2822800) for one day and assigned a study village to conduct water As field measurements. Village workers were responsible for testing the wells for the 50 households using untested wells randomly selected in their assigned village. They were asked to conduct additional tests to locate a nearby low-As well if the well used by one of the 50 assigned households turned out to be unsafe. After testing, a green or red color placard was placed on each well based on compliance with the Bangladesh standard for As in drinking water.

### Field water arsenic measurement

The Hach EZ kit requires the addition of 2 prepackaged reagents, sulfamic acid and zinc powder, into a reaction bottle containing a 50 mL water sample. These reagents produce arsine gas if As is present. This arsine gas is trapped on a reaction strip impregnated with mercuric bromide. The yellow to brown color of the strip is then compared to the reference scale provided by the manufacturer. The scale indicates the intensity of the color expected for As concentrations of 0, 10, 25, 50, 100, 250, and 500 μg/L. A 40 min reaction period was used in this study rather than the 20 min recommended by the manufacturer because a previous study showed that the increased reaction period reduced inconsistencies relative to the Bangladesh As standard in the 50-100 μg/L range [18]. The kit has an optional step to eliminate interference by hydrogen sulfide; this was excluded because sulfide levels in Bangladesh are generally too low to cause interference [18].

### ICP-MS analyses

Water samples were collected in 20 mL acid-washed bottles while the wells were tested in the field. The samples were acidified to 1% with high-purity Optima HCl at least 48 h before analysis. This has been shown to ensure re-dissolution of any As that could have adsorbed to precipitated Fe oxides. Water samples were then diluted 1:10 in a solution spiked with ^73^Ge for internal drift correction and analyzed for As by high-resolution inductively-coupled plasma mass spectrometry (HR ICP-MS), which eliminates the isobaric interference with ArCl. Further details are provided elsewhere [18,19]. The detection limit of the method for As is typically < 0.2 μg/L, estimated here by multiplying the As concentration corresponding to the blank by a factor of 3. The long-term reproducibility determined from consistency standards included with each run averaged 4% (1-sigma) in the 40-500 μg/L range.

### Ethics section

The study protocol was approved by the Columbia University Medical Center Institutional Review Board and the Bangladesh Medical Research Council. Informed consent was obtained from all study respondents.

## Results

### Household drinking water survey

**Figure 3:**
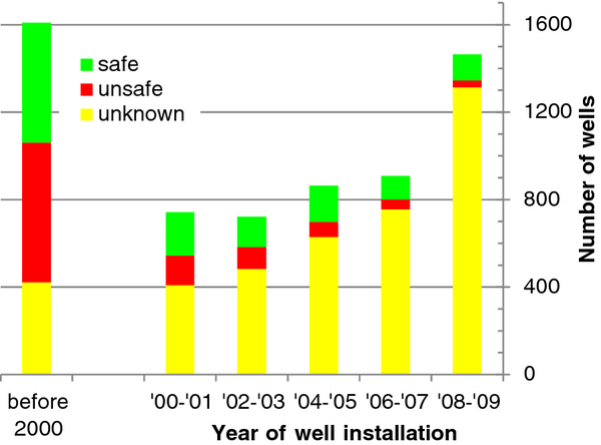
**Status and year of installation of wells reported by 6649 households residing in 26 villages of Singair upazilla**.

Approximately 60% (3989) of respondents interviewed for the household drinking water survey were able to recall the depth of their current primary drinking water source, and 95% (6310) could recall the year of well installation. More than two-thirds of the wells were reported to have been installed within the past 10 years (Figure 3). The rate of installation within each 2-year period increased over the past 10 years, but particularly so during the last 2 years. The reported well depths ranged from 12 to 1400 ft, with a median of 75 ft. When the median of reported depths for wells installed since 2000 is subdivided by year of installation, there is no appreciable change in well depth over time. For example, no two years differed in median well depth by more than 5 ft. Each year is represented by at least 90 values.

Of the 6646 respondents interviewed, 3739 (56%) reported that their well had not been tested for As, 2424 (37%) reported that their well had been tested, and 483 (7%) reported that they did not know whether their well had been tested. Of the tested wells, 1053 (43%) were reported to be safe relative to the Bangladesh As standard of 50 μg/L, 868 (36%) were unsafe, and for 444 (18%) the As status of the well was unknown. Ninety-five percent of the wells that were tested no longer had visible labeling of the As status of the well (i.e., green for safe or red for unsafe). The proportion of untested wells within individual villages ranged from 46 to 83% (Figure 1).

When considering the proportion of untested wells by year of well installation, there is a significant increase over time (p < 0.001 by ANOVA) (Figure 3). For example, 25% of wells installed before 2000 were untested, while roughly 90% of wells installed in the year prior to the survey were untested. Each year is represented by at least 80 values; wells installed in the years prior to 2000 were collapsed to reduce the likelihood of recall bias. A randomly selected subset of 698 households with untested wells were also asked if they knew where a drinking water source considered safe with respect to As was located near their home. Less than 13% (89) of these respondents knew where such a water source was located.

Within the subset of wells that were tested, the proportion of unsafe wells changed over time. Considering two-year intervals for robustness, a significant decrease in the proportion of unsafe wells over time is observed: 54% of wells installed prior to 2000 were unsafe compared to 21% of wells installed between 2008-2010 (p < 0.001 by ANOVA) (Figure 3).

### Arsenic testing intervention

**Figure 4:**
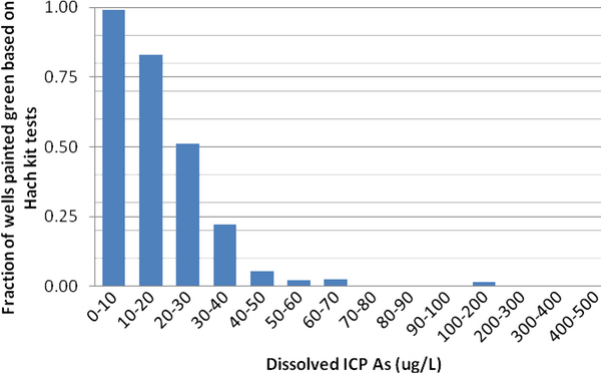
**Results obtained by village workers using the Hach EZ kit with a 40 min reaction time relative to the Bangladesh standard of 50 ug/L for As in drinking water**.

Groundwater As concentrations determined by HR ICP-MS are used as the reference for evaluating the performance of the field kit deployed by village workers. The HR ICP-MS data indicate that As concentrations in the sample range from 0.1 to 437 μg/L, with a median of 54 μg/L. Following the standard interpretation of kit results, a reading above 50 μg/L classifies a well as unsafe relative to the Bangladesh standard for As in drinking water. According to this criterion, the EZ kit underestimated the As content of groundwater relative to these two thresholds for only 4 out of a total 795 samples (Table 1). At the same time, the EZ kit overestimated the As content of groundwater relative to the Bangladesh standard for 163 out of 795 samples. For the vast majority of the overestimates relative to Bangladesh standard, As concentrations were in the 10-50 μg/L range (Figure 4).

**Table 1:** Comparison of Laboratory and Field Kit Results for 795 Wells Tested by Village Testers

ICP-MS As Results	≤ 10 μg/L	10-50 μg/L	50-100 μg/L	> 100 μg/L
**N**	198	292	166	139

**Field Kit Incorrect** **Relative to 10 μg/L**	43	9	0	1

**Field Kit Incorrect** **Relative to 50 μg/L**	2	161	2	2

## Discussion

The largest As testing program in Bangladesh was the BAMWSP survey, conducted between 2001 and 2004 [17]. That survey tested and labeled for As nearly half of the country's 10 million tubewells [16,20]. Thus, in many regions it has been more than six years since the nationwide testing program was conducted. In a study conducted in Araihazar, Bangladesh, it was found that the number of tubewells approximately doubles every two years [21]. If this is the case in other As affected areas, this would imply that the majority of wells in the country are untested for As. In a 2009 national survey conducted by UNICEF and the Bangladesh Bureau of Statistics, it was found that 44% of tubewells in the country were untested [13]. Although there have been many attempts by NGOs and government agencies to provide access to As testing services, many households continue to collect water from untested wells [10,22-28].

Our results from Singair indicating that more than 80% of the tubewells installed during the past 6 years are untested for arsenic is alarming, but not inconsistent with previous observations. The distribution of well ages may provide some evidence for the reason underlying the continuing installation (Figure 3). Unless there is a recall bias, there is no reason to believe the rate of well installation really was actually higher during the past 2 years compared to the four previous 2-year intervals. The apparent sudden increase might suggest instead that a significant fraction of wells are abandoned within the first two years of installation, as suggested by observations elsewhere in Bangladesh [15].

**Figure 5:**
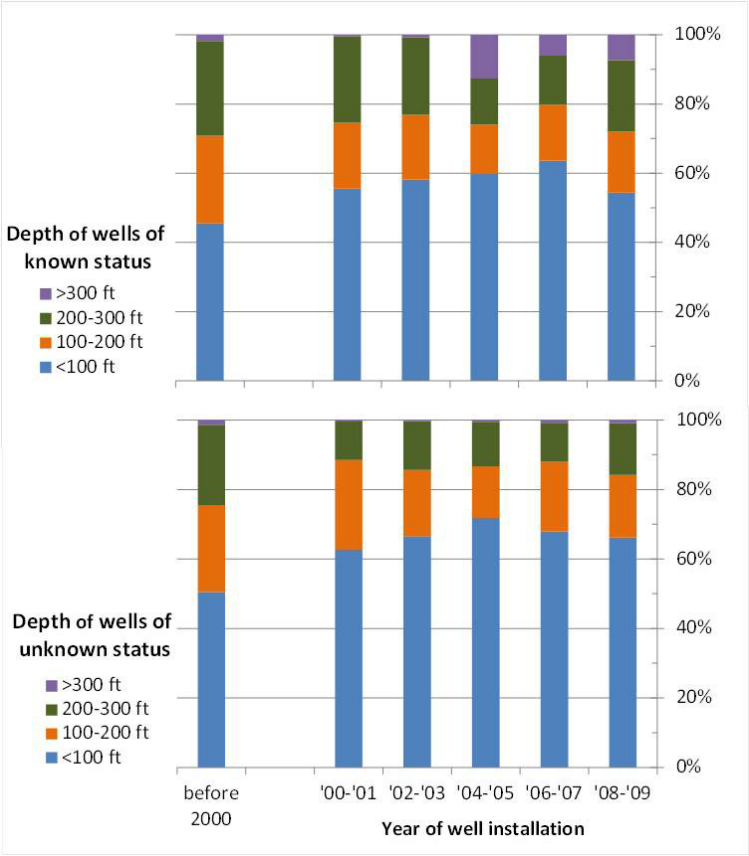
**Comparison of depths of wells of known (1325 households) and unknown status (2648 households) as a function of installation year in 26 villages of Singair upazilla**.

Beyond the first ~100 ft (30 m), the concentration of As generally decreases with depth in aquifers of Bangladesh and there is no reason to believe this wouldn't apply to Singair. The decreasing proportion of unsafe wells within the subset of tested wells over time may therefore at first sight seem surprising given that the depth of wells has not changed. Comparison of trends in well depth over time suggests a possible explanation when tested and untested wells are distinguished (Figure 5). About 25% of the tested wells installed over the past decade in Singair were > 200 ft deep whereas this is the case for only 15% for wells of untested wells. The difference is even more striking for wells > 300 ft deep, typically community wells primarily installed by NGOs or the government. The proportion of deep wells has increased markedly within the group of tested wells whereas very few such wells were untested. This suggests that deep wells installed by NGOs and the government, as well as a sizeable fraction of wells > 200 ft presumably installed by relatively wealthy households, are tested while the shallower wells are not. The trend towards a greater proportion of safe wells within the tested subgroup probably reflects this bias rather than more effective targeting of safe aquifers by all new installations.

**Figure 6:**
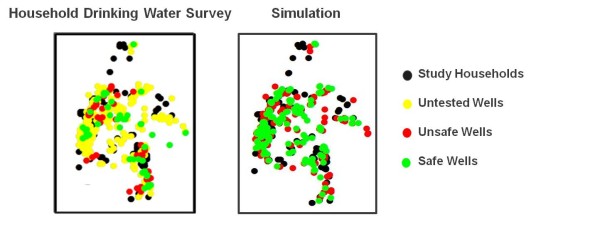
**Close-up view of the spatial distribution of well status in 3 study villages based (a) on the survey and (b) on one of the 10 simulations**.

**Figure 7:**
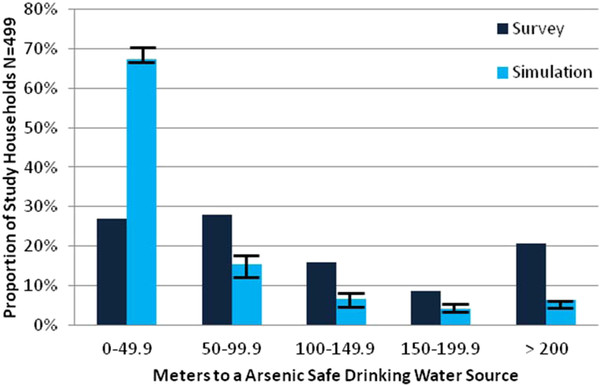
**Distribution of distances to the nearest well for a subset of 499 study households residing within villages where the location of every well was determined**. The area includes 299 unsafe wells, 294 safe wells, and 1208 untested wells. The survey calculation considers wells known to be safe only; the simulation randomly assigned a safe/unsafe status to the wells of unknown status reflecting the 50/50 proportion throughout the study area. Vertical error bars indicate the range in the proportion of distance categories observed for ten such simulations.

To quantify the impact of a growing proportion of untested wells on access to As safe drinking water relative to the Bangladesh As standard as determined by BAMWSP testing, the distance to the nearest well known to be safe from previous testing was calculated for a subset of 499 study households located within 10 villages for which the position of both wells and study households was known (Figure 6). This calculation shows that only 27% of households reside within 50 m of an As safe well and another 28% within 50-100 m of a low-As well (Figure 7).

Previous work has shown that households rarely switch to a private low-As well if it requires traveling more than 100 m each way several times a day [15,17]. If the nearest safe well is within 50 m, the well switching rate in one study area was shown to be 68%, while if the nearest safe well was greater than 150 m away the well switching rate declined to 44% [15]. Ten stimulations were conducted to estimate the potential impact of testing all the untested wells in the same area by randomly assigning a status to untested wells based on the proportion of safe and unsafe wells observed in the study area (50%). The average of all simulations for each distance category shows that testing all of the untested wells within the study area could increase the proportion of study households living within 50 m of an As safe drinking water source from 27 to 67%, and decrease the proportion of households living greater than 100 m away from an As safe drinking water source from 45% to 17%. Collectively, these findings indicate that renewed As testing could significantly reduce exposure.

The results obtained by village workers using the EZ kit and an extended reaction time of 40 min are encouraging and consistent with previous observations [18]. The increased reaction time markedly reduces the number of wells for which the As content is underestimated relative to the standard to increase, but there is clearly a trade-off. The longer time also causes the number of wells incorrectly classified as unsafe relative to the guideline to increase. This reduces the number of wells that a household with an unsafe well could switch to. Given the growing evidence of significant health effects of As exposure in the 10-50 μg/L range, on the other hand, overestimates are clearly preferable to underestimates of the As content of well water [29-31].

The WHO guideline is currently not applied in Bangladesh but our results show that [32,33], using a 40 min reaction time, the Hach EZ kit underestimated the As content of 10 out of 795 wells relative to the 10 μg/L threshold (Table 1). The Hach EZ kit also overestimated the As content of wells for 43 wells relative to the WHO guideline.

## Conclusions

Our household drinking water survey confirmed that there is an urgent need for water As testing in affected areas of Bangladesh. A simple spatial simulation based on the observations shows that testing of wells of unknown status is likely to significantly reduce As exposure by providing information on low As drinking water sources available to households. Our evaluation of the Hach EZ kit using a 40 min reaction time shows that trained village workers will in the vast majority of cases correctly classify wells relative to the current Bangladesh standard for As in drinking water, and could even do so relative to the WHO guideline.

## Abbreviations

As: Arsenic; BAMWSP: Bangladesh arsenic mitigation water supply project; ICP-MS: Inductively-coupled plasma mass spectrometry; LDEO: Lamont Doherty Earth Observatory; NGO: Non-governmental organization; WAs: Water arsenic; WHO: World Health Organization

## Competing interests

The authors declare that they have no competing interests.

## Authors' contributions

CMG directed the field study, performed the statistical analysis, and wrote the manuscript. AVG and JHG directed the studies and revised the manuscript. JM was involved in the laboratory analysis of the water samples collected and revised the manuscript. All authors read and approved the final manuscript.
